# Lung Remodeling in a Mouse Model of Asthma Involves a Balance between TGF-β1 and BMP-7

**DOI:** 10.1371/journal.pone.0095959

**Published:** 2014-04-29

**Authors:** Camila Leindecker Stumm, Erik Halcsik, Richardt Gama Landgraf, Niels Olsen Saraiva Camara, Mari Cleide Sogayar, Sonia Jancar

**Affiliations:** 1 Department of Immunology, University of Sao Paulo, Sao Paulo, SP, Brazil; 2 Department of Biochemistry, University of Sao Paulo, Sao Paulo, SP, Brazil; 3 Department of Biological Sciences, Federal University of Sao Paulo, Diadema, SP, Brazil; 4 Division of Nephrology, Federal University of Sao Paulo, Sao Paulo, SP, Brazil; French National Centre for Scientific Research, France

## Abstract

A key event in chronic allergic asthma is the TGF-β-induced activation of fibroblasts into α-SMA-positive myofibroblasts which synthesize type-I collagen. In the present study we investigated the effect of the anti-fibrotic molecule BMP-7 in asthma. Balb/c mice were immunized i.p. with ovalbumin in alum and challenged every 2 days with ovalbumin aerosol (two or six challenges for acute and chronic protocols, respectively). The lung was evaluated for: α-SMA and type-I collagen by immunohistochemistry; BMP-7 and TGF- β1 gene expression by qRT-PCR; type-I collagen and Smads 2 and 3 by immunoblotting; mucus by PSA staining. Type-I collagen around bronchi, α-SMA, mucus secretion, TGF- β1 and BMP-7 gene expression were all increased in asthma. The TGF- β1/BMP-7 ratio was higher in the chronic group and correlated with higher levels of collagen. Fibroblasts isolated from asthmatic and healthy lungs produced type-I collagen upon stimulation with TGF- β1 via phosphorylation of Smad-2, Smad-3. Pre-treatment of the fibroblasts with BMP-7 reduced collagen production and Smads phosphorylation. Intranasal treatment of asthmatic mice with recombinant BMP-7 during the immunization protocol reduced lung inflammation and type I collagen deposition. These results suggest a protective role for BMP-7 in lung allergic inflammation, opposing the pro-fibrotic effects of TGF- β1.

## Introduction

Allergic asthma is characterized by chronic inflammation of the airways, bronchial hyper-responsiveness, and several structural changes in the lung, collectively termed airway remodeling [1], [2]. It is a Th2-mediated inflammation that involves infiltration of effector T lymphocytes and eosinophils. Products of these cells' activation and degranulation ultimately damage the parenchyma and degrade the extracellular matrix (ECM) components. During this injury, growth factors, which are primarily secreted in their inactive form, bind to the ECM and are released by proteolysis into their active form. TGF- β1 is one of these growth factors, being a key mediator of fibrosis [3], [4]. TGF- β1 induces the differentiation of fibroblasts into myofibroblasts, which are the effector cells in many fibrotic lung diseases, including asthma [5]. The myofibroblast is characterized by expression of α-SMA, and by intensive synthesis of type I collagen, associated with increased proliferation, all key events in wound healing [6], [7]. Specifically in asthma, the characteristic structural changes which occur in the lung are: thickening of the bronchial smooth muscle wall, high number of infiltrating cells, and deposition of an abnormal amount of ECM components beneath the bronchial epithelium. These alterations end up replacing the normal lung parenchyma by scar tissue, diminishing the gas exchange surface and, consequently, determining the decreased respiratory capacity of the patients. Although it has already been established that fibrosis is due to an imbalance in the regulation of the healing process, the elements involved in this deregulation are still not well characterized.

Recently, much attention has been given to the capacity of the tissue to counteract the injury through upregulation of the so-called cytoprotective response. BMP-7, a member of the TGF-β superfamily, was originally described as being involved in the development and homeostasis of several tissues and organs [8]. Further studies showed that it is also involved in the regulation of a wide spectrum of cell functions, such as proliferation, differentiation, and apoptosis [9], [10]. In renal disease, BMP-7 was shown to act as an anti-fibrotic factor, counteracting the actions of TGF-β [11]. TGF-β and BMP-7 signal through independent Smad proteins [12], [13].

In the present study, we investigated the role of these two molecules in lung remodeling in murine asthma, and the mechanisms underlying collagen production by isolated lung fibroblasts.

## Methods

### Reagents

Grade V ovalbumin (OVA) was purchased from Sigma-Aldrich (St. Louis, MO). Bovine serum albumin (BSA) was obtained from US Biological (Swampscott, MA). Aluminum hydroxide (Rehydragel HPA) was obtained from Reheis Inc. (Berkeley Heights, NJ). Dulbecco's modified Eagle's medium (DMEM) and penicillin/streptomycin were obtained from Invitrogen (Carlsbad, CA). Fetal bovine serum (FBS) was purchased from Gibco (Grand Island, NY). TGF-β1 was purchased from Abcam (Cambridge, UK) and the ELISA kit for TGF-β1 from eBioscience (San Diego, CA). The rhBMP-7 used in the *in vitro* experiments was purchased from Gibco (cat # PHC7204), while the rhBMP-7 used in the *in vivo* experiments was a kind gift from Dr. Mari Sogayar from the Institute of Chemistry, University of São Paulo, Brazil [14]. The biological activity of the rhBMP-7 used in our *in vivo* experiments was tested in mouse C2C12 cells (ATCC CRL-1772), proving its activity in mouse cells [14]. In addition, the biological activity of the commercially available BMP-7 used in this work was also tested in murine ADTC5 cells, as per product analysis sheet. Enhanced chemiluminescence (ECL) reagent was obtained from Thermo Scientific (Waltham, MA). Antibodies used for immunoblotting were as follows: anti-rabbit collagen I (AbCam, Cambridge, MA.), anti-β-actin (Sigma-Aldrich). The anti-phospho-p38 MAPK and anti-p38 MAPK; anti-phospho-Smad2, anti-phospho-Smad3 and anti-Smad2/3; anti-phospho-Erk 1/2 and anti-Erk ½ were from Cell Signaling Technology, Beverly, MA. The secondary antibodies used in developing the Western blots were obtained from Sigma-Aldrich and Cell Signaling Technology.

### Animals

Male Balb/c mice weighing 20–25 g, 6–8 weeks old, from our own animal facilities were housed in a room with a 12 h light–dark cycle with water and food *ad libitum*. Animal care and research protocols were in accordance with the principles and guidelines adopted by the Brazilian College of Animal Experimentation (COBEA) and this project was approved by the Ethical Committee for Animal Research of the Institute of Biomedical Sciences of the University of São Paulo.

### Induction of allergic asthma

**Figure 1:**
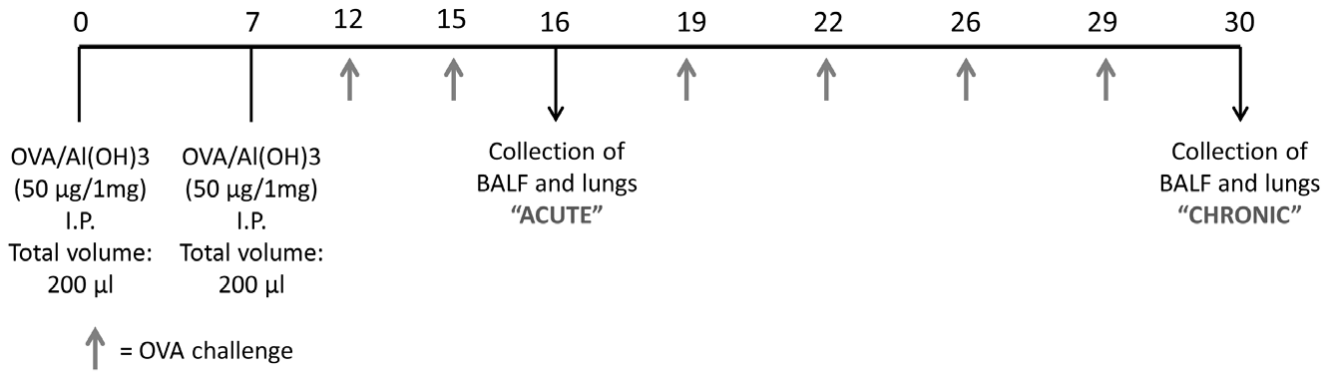
Allergic asthma protocol. Mice were sensitized on days 0 and 7 by an intraperitoneal injection of a mixture containing 50 µg of ovalbumin and 1 mg of Al(OH)_3_ in PBS. Mice were challenged by exposure to an aerosol of OVA for 20 min at days 12, 15, 19, 22, 26, and 29.

Mice were sensitized on days 0 and 7 by an intraperitoneal injection of a mixture containing 50 µg of ovalbumin (OVA; Grade V, Sigma, USA) and 1 mg of Al(OH)_3_ in PBS (a total volume of 0.2 ml). Mice were challenged by exposure to an aerosol of OVA generated by an ultrasonic nebulizer (ICEL US-800, SP, Brazil) delivering particles of 0.5–10 µm diameter at approximately 0.75 cc/min for 20 min at days 12, 15, 19, 22, 26, and 29 (Figure 1). The concentration of OVA in the nebulizer was 2.5% w/v in PBS. The control group consisted of animals immunized as previously described and challenged two or six times with PBS solution. The control animals received two immunizations with OVA/Alum and six challenges with PBS.

### Bronchoalveolar lavage

Animals were sacrificed by asphyxiation, 24 h after exposure to the last aerosol challenge. A tracheal cannula was inserted via a midcervical incision, and the airways were lavaged twice with 1 ml of phosphate-buffered saline (PBS, pH 7.4 at 4°C).

### Total and differential cell counts in the bronchoalveolar lavage fluid

The bronchoalveolar lavage fluid was centrifuged at 170×*g* for 10 min at 4°C, the supernatant was removed, and the cell pellet was re-suspended in 1 ml of PBS. One volume of a solution containing 0.5% crystal violet dissolved in 30% acetic acid was added to nine volumes of the cell suspension. The total number of cells was determined by counting in a hematocytometer. Following cytocentrifugation of the bronchoalveolar lavage fluid, cells were stained with hematoxylin-eosin (Hema 3) for determination of the cell numbers.

### Histology

Lung tissue was harvested and fixed in a 10% buffered-formalin solution and routinely processed for histological inclusion in paraffin. Five-µm thick tissue sections were stained with Periodic Acid of Schiff (PAS) for visualizing mucus and with Masson's Trichrome for visualizing collagen.

### Immunohistochemistry

Tissue sections (5-µm thick) were deparaffinized, hydrated, and submitted to antigenic retrieval by incubation in a 10 mM sodium citrate buffer at 90°C for 20 minutes. Sections were treated with 3% H_2_O_2_ in PBS for 30 minutes to block endogenous peroxidase activity. Non-specific staining was blocked by incubating the sections for 30 minutes with 10% bovine serum albumin in PBS. Primary antibodies were incubated overnight at 4°C in a concentration of 1∶200 in 0.3% Tween 20 in PBS. Sections were thoroughly washed with PBS and then incubated with biotin-conjugated goat anti-rabbit IgG (Vector, USA) for 1 hour at room temperature. After washes in PBS, sections were incubated in streptavidin-peroxidase ABC kit (Vector, USA) for 1 hour at room temperature. Peroxidase was visualized using 0.03% 3,3′-diaminobenzidine in PBS with 0.08% H_2_O_2_. Sections were counterstained with Mayer's Hematoxylin, dehydrated in ethanol, and mounted in VectaMount mounting medium (Vector, USA).

### Morphometric analysis

Morphometric analysis was performed using a Nikon DXM 1200c digital camera, and Nikon NIS – Elements AR 2.30 software. The area of positivity was measured (in µm^2^) in the maximal number of bronchioles per slide and the area of each bronchiole was normalized by the average of three different measurements of the diameter of the same bronchiole to rule out the influence of the caliber of the bronchiole in the extent of collagen deposition. Results are expressed as the mean of total area/diameter for each animal, and as mean values ± standard errors with comparisons between groups performed by analysis of variance (ANOVA). *P*<0.05 was considered statistically significant.

### Determination of the concentration of TGF-β1 and BMP-7

TGF-β1 and BMP-7 were measured by ELISA in the BAL supernatants according to the manufacturer's instructions.

### Real-time RT-PCR

Lung tissue was collected, washed in sterile PBS, and frozen in liquid nitrogen, and then stored at −80°C. Total RNA was extracted with Trizol Reagent (Invitrogen, USA) and the concentration of RNA was determined by spectrophotometer reading at absorbance 260 nm. cDNAs were synthesized by reverse transcriptase MML-V (Promega, USA). Real-time PCR was performed using the semi-quantitative SYBR Green assay (Applied Biosystem, USA) using specific primers for collagen I, BMP-7, and TGF-β1, and HPRT as the housekeeping gene. Primers were designed according to the RNA sequence published on GenBank. Primer sequences were as follows: BMP-7 sense: TTT GAC ATC ACA GCC ACC AGC AAC, anti-sense: ATG AAC CTC CGT GGC CTT GAA GAA; Collagen I sense: TGG CCA AGA AGA CAT CCC TGA AGT, antisense: ACA TCA GGT TTC CAC GTC TCA CCA; TGF-β1 sense: AAC TAT TGC TTC AGC TCC ACA GAG; anti-sense: AGT TGG ATG GTA GCC CTT G; HPRT sense: CTC ATG GAC TGA TTA TGG ACA GGA C; anti sense  = GCA GGT CAG CAA AGA ACT TAT AGC C. The amount of the target gene was normalized first to the endogenous reference (HPRT) and then relative to a calibrator (control animal), using the 2^−ΔΔCt^ method [15]. Steady-state mRNA levels are thus expressed as an n-fold difference relative to the calibrator. Analyses were performed with the Sequence Detection Software 1.9 (SDS). Results are expressed as mean values ± standard errors with comparisons between groups performed by analysis of variance (ANOVA).

### Primary culture of mouse lung fibroblasts

Mouse lungs were perfused via the right ventricle with 5 ml cold PBS and removed under aseptic conditions. The left lung was minced with scissors in DMEM containing 10% FBS and placed in 10 ml of medium in 10-cm diameter tissue culture plates. Fibroblasts were allowed to grow out of the minced tissue and when cells reached 70% confluence, they were passaged following trypsinization. Fibroblasts were grown for 10–15 days (2–3 passages) before being used. The methodology employed here was based on the finding of Moore et al, [16] that following 14 days in culture, the cells are uniformly positive for collagen I protein as evidenced by intracellular flow cytometry analysis and also express mRNA for collagen I, collagen III and fibronectin. A different group [17] has used the exact same fibroblast isolation technique and has described that fibroblasts isolated this way stain positive for vimentin and negative for keratin, and that by transmission electron microscopy they have morphology consistent with that of fibroblasts. Our group has previously done lung fibroblast purification and culture according to this methodology [18].

### Immunoblot analysis

For collagen I analysis, the cells were plated, serum-starved overnight, and then treated for 18 h with TGF-β1 (5 ng/ml) alone or in combination with BMP-7 (300 ng/ml). For phosphorylated proteins, cells were treated for 15 minutes with the same concentrations of TGF-β1 and BMP-7. Cells were washed in PBS and disrupted in lysis buffer (PBS containing 1% Nonidet-P40, 0.5% sodium deoxycholate, 0.1% SDS, 2 mM orthovanadate, and Roche protease cocktail inhibitor). Equal amounts of lysate protein were separated by SDS-PAGE and transferred to nitrocellulose membranes. Membranes were blocked in 5% nonfat milk in Tris-buffered solution with 0.1% Tween. They were then incubated overnight at 4°C with the respective primary antibodies against collagen I, phospho-p38 MAPK, phospho-Smad2, and phospho-Smad3 (1∶1000 in 2% BSA/0.02% sodium azide in TBST). Proteins of interest were detected using the ECL enhanced chemiluminescence method. Densitometry of the bands was calculated with DigiDoc1000 (AlphaEase FC software, version 3.2 Beta, AlphaInnotech) and normalized for β-actin or the respective total proteins, which were used as loading controls.

### In vivo treatment with rhBMP-7

**Figure 2:**
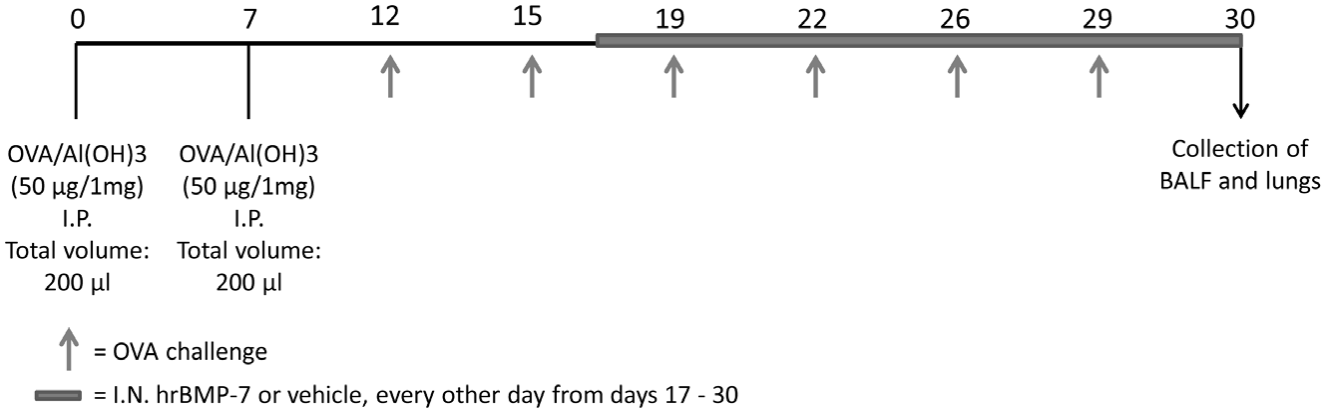
*In vivo* treatment with rhBMP-7. Mice were anesthetized and human recombinant BMP-7 (300 mg/kg in a total volume of 30 µl) in PBSA isotonic buffer (140 mM NaCl, 2,7 mM KCl, 8 mM Na_2_HPO_4_ and 1.5 mM KH_2_PO_4_), was administered intranasally every other day between days 17 and 30 of the chronic asthma protocol.

Mice were anesthetized by an i.p. injection of 100 µl of 5% xylazine/10% ketamine v/v in sterile PBS. Human recombinant BMP-7 (300 mg/kg in a total volume of 30 µl) in PBSA isotonic buffer (140 mM NaCl, 2,7 mM KCl, 8 mM Na_2_HPO_4_ and 1.5 mM KH_2_PO_4_), was administered intranasally every other day between days 17 and 30 of the chronic asthma protocol (Figure 2). Animals in the control groups received the same volume of the vehicle by the same route and timing. Lungs were processed for histology, stained with Masson's trichrome for collagen, and morphometric analysis was performed as described.

### Data analysis

Data are presented as mean values ±SEM of 5 mice per group in each of 3 independent experiments. Statistical significance was analyzed using GraphPad Prism 5 (version 5.01; GraphPad Software). Significance was assessed by ANOVA and a post hoc Bonferroni test for three or more groups. *P*<0.05 was considered significant.

## Results

### Establishment of the chronic asthma protocols

Balb/c mice were immunized with two i.p. inoculations of OVA/alum and subjected to six exposures to aerosol of OVA. One day after the last exposure, they were euthanized and the lungs processed for evaluation of cell infiltration, mucus, collagen, and α-SMA. The number of cells in the bronchoalveolar lavage fluid increased from 18.6±1.2 in the control to 79.6±16.5 in asthmatic lungs. This increase was due to migration of eosinophils and neutrophils, which amounted to 70 and 26% of the cells, respectively.

**Figure 3:**
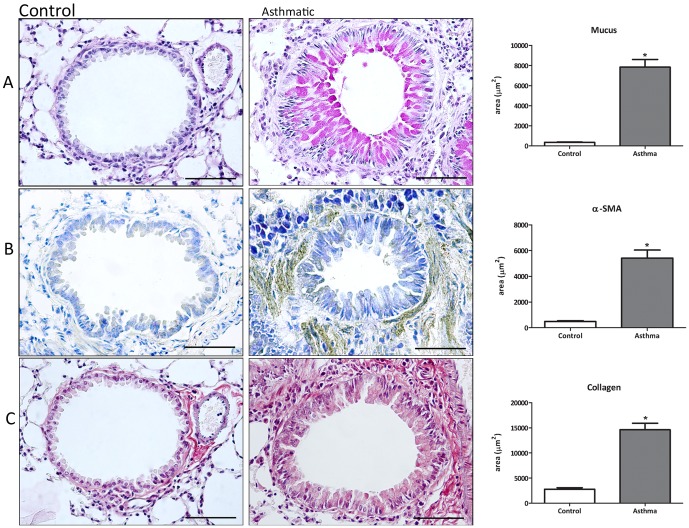
Fibrosis markers in the asthmatic lung. Lung sections were taken from animals with asthma (6 challenges), stained with PAS for mucus (A) or prepared for immunohistochemical and morphometric analysis of α-SMA (B) and collagen type I (C). Data are representative of 5 animals in each of 3 independent experiments. Bars  = 50 mm. *p<0.05 comparing with control group.

Mucus production was evaluated in lung histological sections stained with PSA. As shown in Figure 3A, the morphometric analysis shows a significantly higher level of mucus in the asthmatic group, when compared with the control group.

Alpha smooth muscle actin (α-SMA) expression has been extensively used as a marker of fibroblast differentiation into its activated state, the myofibroblast. Expression of α-SMA was followed by thickening of the bronchial muscular wall, increasing around the bronchi of animals that were exposed to six allergen challenges, when compared to the control group (Figure 3B). These results were confirmed by morphometric analysis.

The α-SMA-positive myofibroblasts actively synthesize ECM components. Initially we measured peribronchial collagen in lung sections stained with Picrosirius. We found enhanced deposition around small bronchi in the asthmatic group, when compared to the control group (data not shown). This staining does not differentiate between different types of collagen. We then measured type I collagen, the main collagen isoform produced by fibroblasts in many fibrotic processes. By immunohistochemistry, we found increased type I collagen in the sub-epithelial region of small bronchi in the asthma group. Morphometric analysis showed that type I collagen deposition was significantly higher in the asthma group when compared to the control group (Figure 3C). Taken together, these results show that this asthma model proves effective in generation en effector response, where there is evident lung tissue remodeling with a fibrotic component around the airways.

### The ratio TGF-β1/BMP-7 as a tool to evaluate the intensity of lung fibrosis

**Figure 4:**
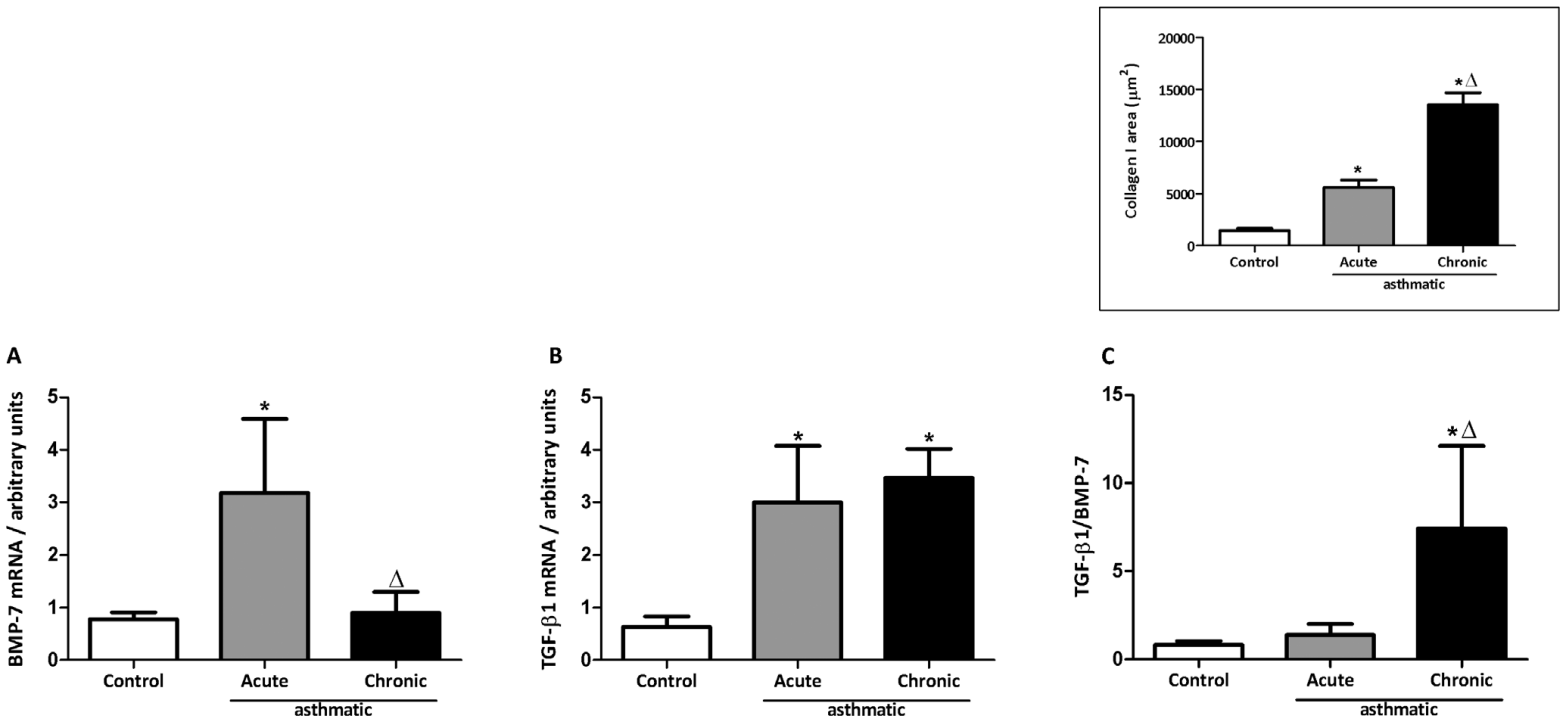
TGF-β and BMP-7 mRNA expression in lung tissue. BMP-7 (A) and TGF-β (B) mRNA in whole lung preparations. The TGF-β/BMP-7 ratio was calculated (C). HPRT was used as housekeeping gene. Data are representative of 5 animals in each of 3 independent experiments. *p<0.05 comparing with control group; Δ p<0.05 comparing acute with chronic groups. In the insert, collagen-I (area/um^2^).

TGF-β1 is the prototypic pro-fibrotic cytokine and its signaling is inhibited by BMP-7. As expected, TGF-β1 was elevated in the BALF of asthmatic animals when compared to controls. Treatment with BMP-7 300 mg/kg reduced TGF-β1 levels in the BAL fluid, showing a further regulatory role of BMP-7, not only over TGF-β1 signaling but possibly also synthesis or secretion. Levels of TGF-β1 were 61.10±9.432 pg/ml in the controls, 386.3±54.3 pg/ml in the asthmatic animals and 200.0±13.56 ng/ml in the asthmatic animals treated with BMP-7. Increased collagen, as seen in Figure 3, correlates with predominance of TGF-β1 over BMP-7. The mRNA expression of these molecules, analyzed in homogenates of whole lung, showed similar levels of TGF-β1 in the acute and chronic asthma groups, but the expression of BMP-7 was much higher in the acute asthma group (Figures 4A and B). When the TGF-β1/BMP-7 ratio (Figure 4C) was analyzed, acute asthma correlated with a low ratio whereas chronic asthma correlated with a higher ratio. Moreover, there was a direct correlation between the TGF-β1/BMP-7 ratio and the levels of collagen deposition (see insert of Figure 4). These results suggest that the TGF-β1/BMP-7 ratio could be used to predict the intensity of lung fibrosis.

### BMP-7 inhibits TGF-β1-induced collagen synthesis by lung fibroblasts

**Figure 5:**
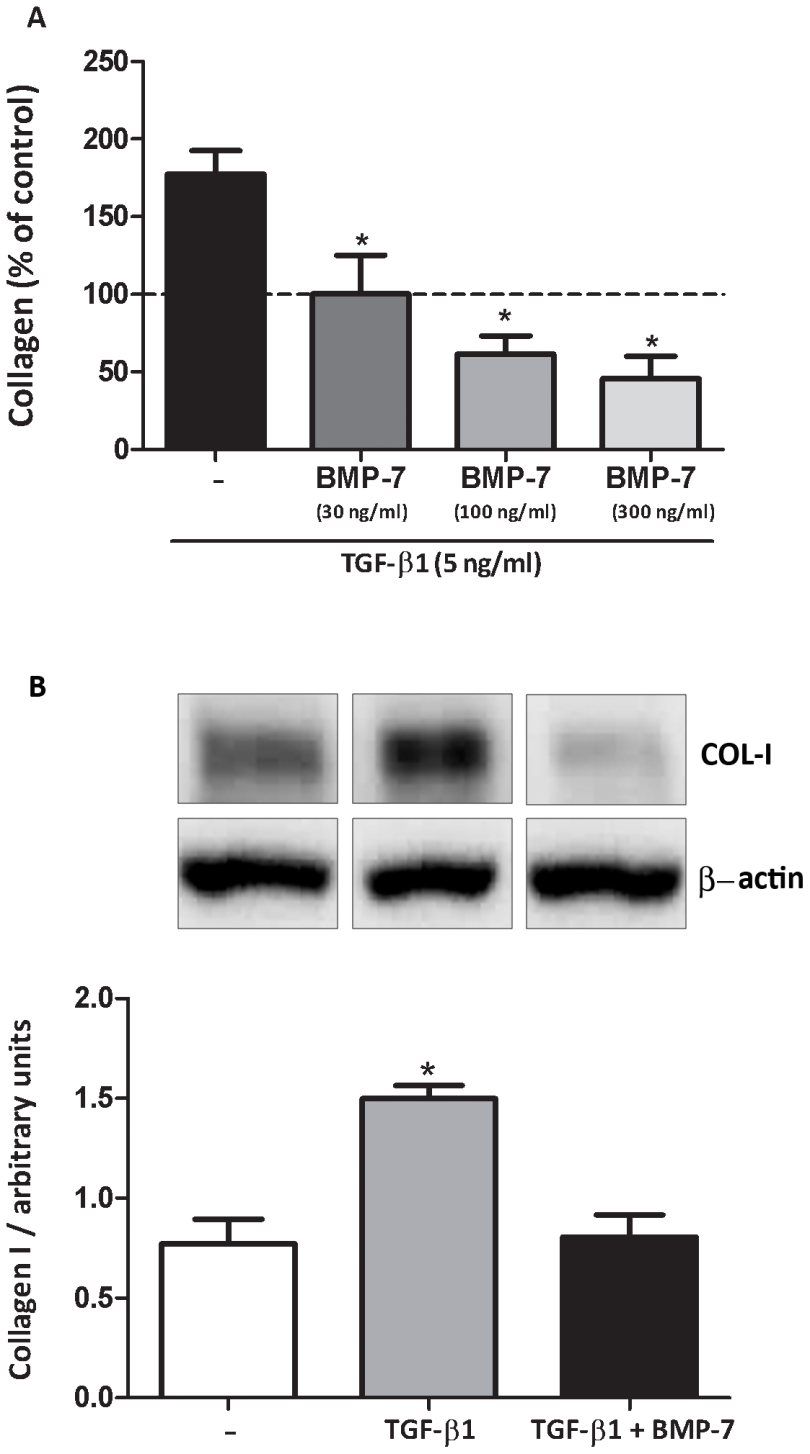
BMP-7 inhibits TGF-β1-induced synthesis of collagen-I by lung fibroblasts. Mouse lung fibroblasts from control animals (A) were stimulated with TGF-β1 (5 ng/ml) alone or in combination with BMP-7 (30, 100 ou 300 ng/ml) during 18 hours. Mouse lung fibroblasts from asthmatic animals (B) were treated with TGF-β1 (5 ng/ml) alone or in combination with BMP-7 (300 ng/ml). Type I collagen was analyzed by western blot and normalized by β-actin expression. Results are presented as percentage of the control or as arbitrary units. Data are representative of 5 animals in each of 3 independent experiments. *p<0.05 comparing with the control group.

Fibroblasts were isolated from the lungs of healthy mice and incubated with TGF-β1 (5 ng/ml) alone or in combination with BMP-7 (30, 100, and 300 ng/ml). TGF-β1 increased type I collagen expression, which was reversed, in a dose-dependent fashion, by co-incubation with BMP-7 (Figure 5A). Similar results were obtained when fibroblasts were isolated from asthmatic lungs (data not shown).

**Figure 6:**
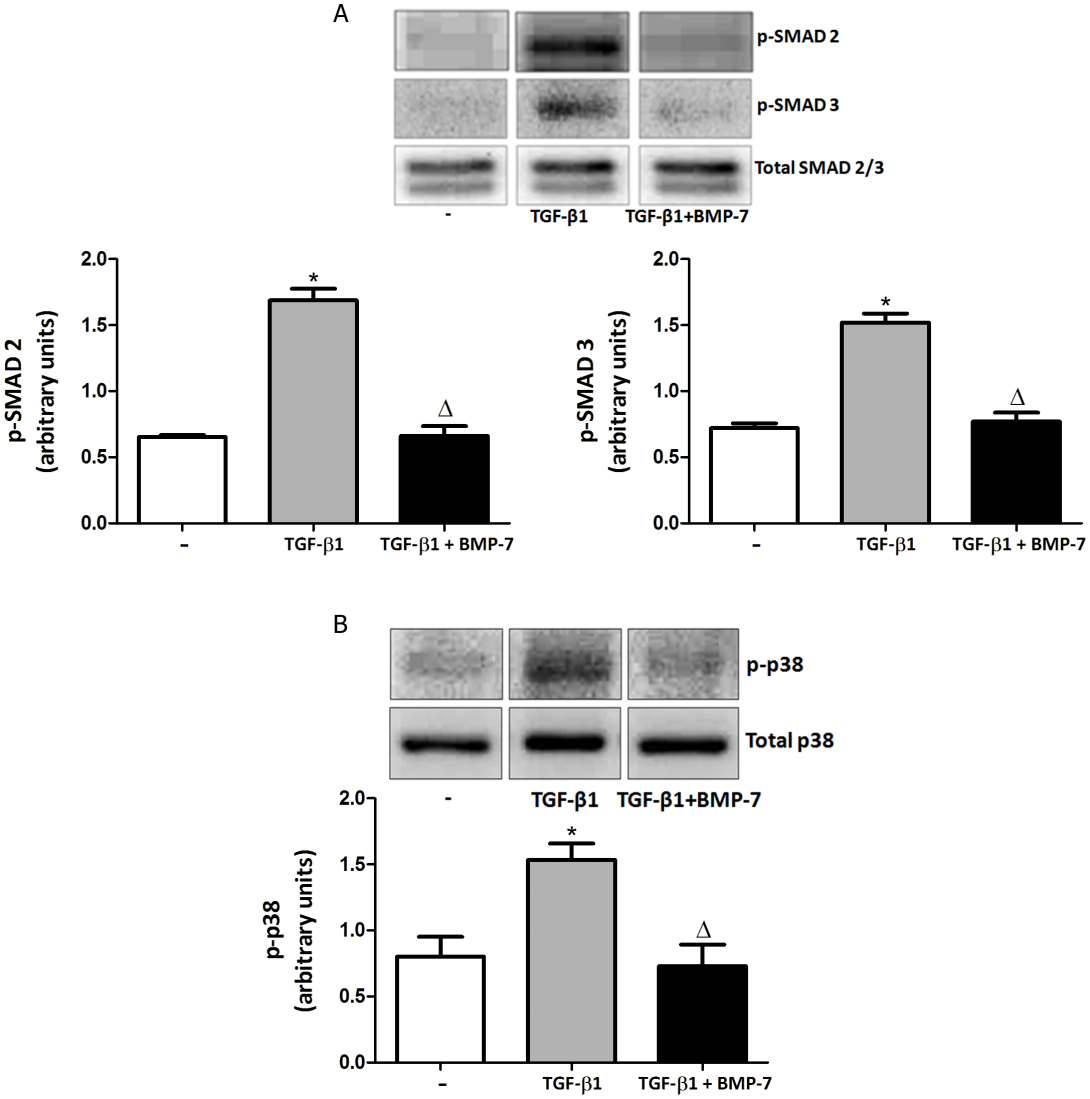
BMP-7 inhibits SMADs phosphorilation in mouse lung fibroblasts from chronic asthmatic mice. Mouse lung fibroblasts were treated with TGF-β1 (5 ng/ml) alone or in combination with BMP-7 (300 ng/ml). After 15 min, samples were analyses for SMAD 2 and 3 or p-p38 expression. Proteins were analyzed by western blot and normalized by their respective total proteins. Data are representative of 5 animals in each of 3 independent experiments. *p<0.05 comparing with the control group; Δ p<0.05 comparing TGF-β1-treated with TGF-β1/BMP-7-treated groups.

To investigate which signaling pathways activated by TGF-β1 are inhibited by BMP-7, we chose the classic Smad-dependent pathway (Smad 2 and 3) and the Smad-independent pathway, p38 MAPK, since these signaling pathways have been extensively implicated in fibrosis models and in human diseases involving fibrotic processes. Figure 6 shows that, in fibroblasts isolated from chronic asthmatic lungs, TGF-β1 induced the phosphorylation of Smads 2 and 3 (Figure 6A), which was partially inhibited by BMP-7. TGF-β1 also increased p-38 phosphorylation (Figure 6B), which was similarly prevented by BMP-7. Similar results were obtained with fibroblasts isolated from healthy lungs (data not shown). We believe that these results indicate that in mouse lung fibroblasts, as described for hepatic cells, BMP-7 is able to at least partially inhibit TGF-β1 signaling, measured as inhibition of phosphorylation of key signaling components such as p-38 and Smads.

### Treatment of asthmatic mice with rhBMP-7 inhibits lung inflammation and collagen deposition

**Figure 7:**
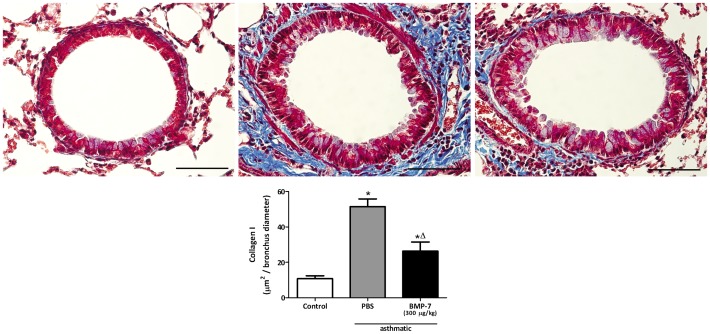
Intranasal treatment with BMP-7 reduces collagen-I expression in chronic asthmatic lungs. Lung slides were stained with Masson's Trichrome (blue) and the morphometric analysis was performed as described in methods. Control (A), Chronic asthmatic (B), Chronic asthmatic treated with BMP-7 (300 µg/kg) by intranasal route (C). Morphometric analysis of the stained area (blue)(D). Data are representative of 5 animals in each of 3 independent experiments. Bars  = 50 µm. *p<0.05 comparing with the non asthmatic control; Δ p<0.05 comparing asthmatic mice treated or not with BMP-7.

**Figure 8:**
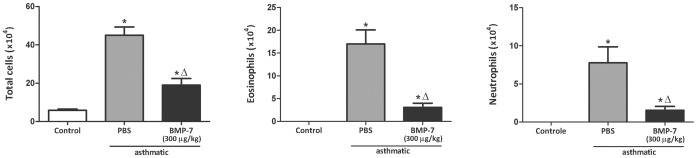
Intranasal treatment with BMP-7 reduces cellular infiltrate in the airways of chronic asthmatic lungs. Total cells in BAL were counted in a hemocytometer. After cytocentrifugation cells were stained with H.E. for determination of eosinophil and neutrophil numbers. Data are representative of 5 animals in each of 3 independent experiments. *p<0.05 comparing with the non asthmatic control; Δ p<0.05 comparing asthmatic mice treated or not with BMP-7.

We showed here that BMP-7 inhibits the TGF-β1-induced collagen production by lung fibroblasts *in vitro*, correlating inversely with the degree of collagen deposition in the lung. Therefore, we sought to investigate whether *in vivo* administration of BMP-7 would reduce lung fibrosis. In the chronic asthma protocol, rhBMP-7 was administered during the fibrogenic phase of asthma by intranasal instillation of 300 µg/kg in a total volume of 30 µl of BMP-7 every other day between days 17 and 30. This treatment markedly reduced collagen deposition around small bronchi (Figure 7) and also reduced cell infiltration in the airways, markedly reducing eosinophils and neutrophils in the bronchoalveolar lavage fluid (Figure 8). These results show for the first time that treatment with BMP-7 is able to attenuate airway fibrosis in the mouse allergic asthma setting.

## Discussion

The persistent inflammation induced by an allergen in sensitized animals is followed by several structural changes in the tissue, known as airway remodeling. Regeneration of an injured tissue involves a tuned balance among molecules that promote or degrade the ECM. Imbalance of this process leads to excessive deposition of ECM proteins, mainly type I collagen, leading to fibrosis and reduction of the lung function [19]. The cellular and molecular mechanisms behind this imbalance are still poorly understood.

In the present study, we first established a model of chronic asthma where OVA-immunized mice were given six OVA challenges, and measured collagen deposition around bronchi and α-SMA as markers of fibrosis. These parameters, as well as mucus secretion, were significantly increased in the asthma model.

As occurs in patients, the chronic exposure to allergens leads to altered remodeling of lung tissue, culminating in airway fibrosis [20]. One of the important events in the remodeling process is the TGF-β1-induced activation of fibroblasts into myofibroblasts, which express α-SMA protein. In the chronic model, we observed an increase in α-SMA expression around small bronchi, which is probably related to local differentiation of fibroblasts into myofibroblasts. In the lungs, the α-SMA-positive cells actively synthesize ECM components, such as type I collagen, and type I collagen was found in the sub-bronchial region. Type I collagen is the main collagen isoform deposited in tissues in many fibrotic processes [19], [21]. To counteract the fibrogenic effect of TGF-β1, several molecules can inhibit its signaling, among them BMP-7. Both TGF-β1 and BMP-7 belong to the same cytokine superfamily, though each of these cytokines presents a unique signaling pathway with different Smads determining opposite effects.

When we compared the expression of these molecules in acute and chronic protocols of asthma, we found that the gene expression for BMP-7 is increased in the acute compared with the chronic group, but the expression of TGF-β1 was similar in both groups. The ratio between these molecules clearly shows a predominance of BMP-7 in the acute model, which has less collagen, and predominance of TGF-β1 in the chronic model, which shows higher levels of collagen. This is in accordance with studies that showed increased fibrosis when BMP-7 signaling is blocked [22]. We propose that the TGF-β1/BMP-7 ratio could be used as a marker of disease severity in asthma, as has been proposed for renal chronic fibrosis [23].

It has been reported that the BMP family counteracts TGF-β1-induced epithelial-to-mesenchymal transition and reverses chronic renal injury [19]. In human lung fibroblasts, BMP-7 partially inhibited myofibroblast-like transformation by TGF-β and BMP-4 reduced TGF-β1-induced extracellular matrix protein production [24]. In our study, mouse lung fibroblasts from control and asthmatic animals were grown in culture and treated with TGF-β1 alone or in combination with BMP-7. Treatment with BMP-7 was effective in inhibiting type I collagen synthesis induced by TGF-β1. The mechanisms exerted by BMP-7 over specific TGF-β1 signaling pathways have not been previously described in mouse lung fibroblasts. In our study, BMP-7 inhibited the TGF-β1-induced phosphorylation of Smad-2 and -3 and p-38.

We then investigated the effect of *in vivo* administration of rhBMP-7 in collagen deposition in the chronic asthma model. Mice were treated with a dose of rhBMP-7 (300 µg/kg) every other day between days 17 and 30. As expected, TGF-β1 was elevated in the BALF of asthmatic animals when compared to controls. Treatment with BMP-7 300 mg/kg reduced TGF-β1 levels in the BAL fluid, showing a further regulatory role of BMP-7, not only over TGF-β1 signaling but possibly also synthesis or secretion. This is in accordance with a previous work, [25] showing that BMP-7 is able to abrogate TGF-β1 activity in renal proximal tubular epithelial cells both by increasing its binding to the extracellular matrix and by inhibiting its generation in vitro. The induction phase of allergic response to OVA was not influenced by BMP-7 treatment. The animals were immunized and challenged and the treatment with BMP-7 began on the 16^th^ day after the first immunization (day 17 of the protocol). At this time, antibodies have been already produced as well as the effector Th2 lymphocytes. At the point where BMP-7 was administered, the intranasal antigen induced effector T lymphocytes migration to the lungs had already happened, with activation leading to the chronic lung inflammation. This window of treatment was chosen because, in our model, it corresponds to the fibrogenic phase, therefore being of therapeutic relevance. Administration of BMP-7 was performed via the intranasal route. This treatment was able to significantly decrease collagen deposition around small bronchi. Similarly, in a model of pulmonary fibrosis induced with asbestos, treatment with BMP-7 decreased the hydroxyproline levels, counteracting its antagonist Gremlin, whose levels are elevated in fibrotic lungs [26]. There is data showing that on a model of hepatic fibrosis, the BMP-7 RNA levels in the liver are the same whether the animals are treated or not with BMP-7. Also, treatment with BMP-7 increases the levels of Gremlin, which is an antagonist that prevents BMP-7's ligation to its receptor [27]. Despite the different variables that can influence the effect of in vivo treatment with BMP-7, our work is the first description of the anti-fibrotic role of BMP-7 in an allergic setting, such as asthma. We speculate that in asthma, an increase in BMP-7 expression occurs after the first exposures to the antigen, aiming to protect the lung from the allergic injury. With persistent exposure to the antigen, an imbalance between these two molecules ensues, with prevalence of TGF-β1, leading to fibrosis.

In conclusion, our work sheds light on the balance between TGF-β1 and BMP-7 in the remodeling process in the lung in a model of allergic asthma and proposes that the TGF-β1/BMP-7 ratio could be used as a marker of disease severity. Moreover, it suggests the possibility of interfering with the fibrotic process in asthma by intranasal instillation of BMP-7.
